# Peroxisome Proliferator-Activated Receptor *γ* Regulates Lipid Metabolism in Sheep Trophoblast Cells through mTOR Pathway-Mediated Autophagy

**DOI:** 10.1155/2023/6422804

**Published:** 2023-11-08

**Authors:** Kexing Hao, Jing Wang, Hengbin Yu, Lei Chen, Weibin Zeng, Zhengrong Wang, Guangdong Hu

**Affiliations:** ^1^State Key Laboratory of Sheep Genetic Improvement and Healthy Production/Institute of Animal Husbandry and Veterinary, Xinjiang Academy of Agricultural and Reclamation Sciences, Shihezi, China; ^2^College of Animal Science and Technology, Shihezi University, Shihezi 832000, China

## Abstract

Peroxisome proliferator-activated receptor gamma (PPAR*γ*) is a key nuclear receptor transcription factor that is highly expressed in trophoblastic cells during embryonic attachment and is accompanied by rapid cell proliferation and increased lipid accumulation. We previously showed that the autophagy pathway is activated in cells after activation of PPAR*γ*, accompanied by increased lipid accumulation. In this study, we used PPAR*γ* agonist rosiglitazone and inhibitor GW9662, as well as autophagy activator rapamycin and inhibitor 3-methyladenine, to unravel the probable mechanism of PPAR*γ* engaged in lipid metabolism in sheep trophoblast cells (STCs). After 12 h, 24 h, and 48 h of drug treatment, the levels of autophagy-related proteins were detected by Western blot, the triglyceride content and MDA level of cells were detected by colorimetry, and the lipid droplets and lysosomes were localized by immunofluorescence. We found that PPAR*γ* inhibited the activity of mammalian target of rapamycin (mTOR) pathway in STCs for a certain period of time, promoted the increase of autophagy and lysosome formation, and enhanced the accumulation of lipid droplets and triglycerides. Compared with cells whose PPAR*γ* function is activated, blocking autophagy before activating PPAR*γ* will hinder lipid accumulation in STCs. Pretreatment of cells with rapamycin promoted autophagy with results similar to rosiglitazone treatment, while inhibition of autophagy with 3-methyladenine reduced lysosome and lipid accumulation. Based on these observations, we conclude that PPAR*γ* can induce autophagy by blocking the mTOR pathway, thereby promoting the accumulation of lipid droplets and lysosomal degradation, providing an energy basis for the rapid proliferation of trophoblast cells during embryo implantation. In brief, this study partially revealed the molecular regulatory mechanism of PPAR*γ*, mTOR pathway, and autophagy on trophoblast cell lipid metabolism, which provides a theoretical basis for further exploring the functional regulatory network of trophoblast cells during the attachment of sheep embryos.

## 1. Introduction

Hormonal regulation of embryonic implantation mediates the gradual establishment of close communication between embryonic trophoblast cells and maternal endometrial cells. These interactions lead to structural and functional remodeling of the embryo. Among these contacts, the nourishing ectoderm is involved principally in the interaction between the embryo and the endometrial epithelium during embryo attachment which guides embryo implantation. This process usually is completed 28-35 days after fertilization in sheep [1]. In contrast, blastocyst production is prolonged during implantation in ruminants and pigs [2]. Progesterone production in sheep depends on the endometrium which provides abundant nutrients and signal regulation molecules that are required for progesterone synthesis. The continuation of progesterone production further stimulates the endometrium to release nutrients into the uterine cavity. Trophoblast cells proliferate and fuse with endometrial cells during this process to accelerate nutritional regulation and promote further progesterone production [3].

The structural and functional remodeling of the placental trophoblast is crucial for rapid growth of the embryo during gestation. Substance metabolism and signal exchange are regulated through marked changes in the proliferation, migration, and invasion of trophoblast cells so as to maintain normal embryo attachment [4]. Fatty acids provide the energy that is needed for rapid progesterone production, ensure the proliferation of trophoblast cells during progesterone synthesis, and provide energy for cells to synthesize and secrete a large number of bioactive factors for intercellular signal transduction [5]. Lipids are the main form of energy storage in animals and are a critical energy source for cellular physiological activity. Lipids are oxidized during pregnancy to produce ATP and are vital precursors of lipid derivatives, such as eicosadienoic acid, that are produced during embryo development [6]. The expression of genes involved in peroxisome formation and fatty acid activation is upregulated during bovine conceptus [7].

Peroxisome proliferator-activated receptor *γ* (PPAR*γ*) is an important regulator of lipogenesis, lipid metabolism, and insulin sensitivity [8]. PPAR*γ* is a nuclear transcription factor containing multiple domains, including an n-terminal transactivation domain (AF1), a highly conserved DNA-binding domain (DBD), and a C-terminal ligand-binding domain (LBD) which has a ligand-dependent transactivation function (AF2) [9]. Different domains of the protein have dissimilar ligand targets, including natural and synthetic ligands. Thiadiazolidinones (TZDs), such as rosiglitazone, pioglitazone, and troglitazone, are synthetic PPAR*γ* ligands which bind to the LBD of PPAR*γ*, activating the AF2 surface to accommodate the coactivators [10, 11]. Among them, rosiglitazone has been widely used in reproductive studies to regulate the activity of PPAR*γ* [12]. In addition to activators, PPAR*γ* has multiple antagonists, and GW9662, which is considered the most potent antagonist, irreversibly binds to PPAR*γ* by covalent modification of Cys285 [13]. PPAR*γ* is highly expressed in the uterine tissue of pigs, placenta of dogs, and placenta cotyledon of ewes during the peri-implantation period [14]. Transcriptome sequencing of bovine embryos revealed that the expression of PPAR*γ* increased significantly at the beginning of progesterone synthesis and continued at a high level throughout the period of progesterone production [7]. Moreover, PPAR*γ* transcription levels in placental trophoblastic cells increased steadily from day 12 to day 17 after fertilization in sheep, and trophoblastic proliferation and lipid metabolism occurred [15]. Inhibition of PPAR*γ* transcription leads to severe developmental retardation of sheep conceptus [16]. However, drug-induced PPAR*γ* expression in sheep during the attachment period upregulated the expression of genes involved in embryo attachment as well as the expression of key genes that are implicated in progesterone production in trophoblast cells [17]. PPAR*γ*-deficient mouse embryos usually lack lipid droplets (LDs) that are present in PPAR*γ*-proficient placenta, and LDs are present in the cytoplasm of trophoblast cells during healthy sheep conceptus [18].

Lipids in cells mainly exist in the form of lipid droplets. Studies have shown that autophagy may play an important role in adipogenesis, and PPAR*γ* is also associated with autophagy [19–22]. Autophagy is a crucial catabolic process during which cytoplasmic contents and subcellular organelles are degraded. TZDs induce neural precursor expressed and developmentally downregulated protein 4 (NEDD4) transcription and protein expression levels by upregulating PPAR*γ* activity in HepG2 hepatocytes, thereby promoting Akt phosphorylation and autophagy [23]. Activation of PPAR*γ* can significantly reduce lung injury and pulmonary edema, inhibit the expression of inflammatory factors, and promote autophagy, while GW9662 can aggravate inflammatory injury and inhibit autophagy [24]. Moreover, activated PPAR*γ* can enhance autophagy through AMPK/mTOR signaling pathway to protect renal tubular cells from hypoxia/reoxygenation injury [25]. Mammalian target of rapamycin (mTOR) is an indispensable kinase in the PI3K/AKT/mTOR signaling pathway and is the principal negative regulator of autophagy. The inhibition of mTOR activity is important for initiating the formation of autophagosomes, and the specific mTOR inhibitor rapamycin activates autophagy [26]. Phosphorylation of mTOR is essential for cell cycle progression, proliferation, and survival. The PPAR*γ* activator pioglitazone protects renal tubular cells from hypoxia and reoxygenation injury by enhancing autophagy through the AMPK/mTOR signaling pathway [25].

In view of the links between PPAR*γ*, mTOR, autophagy, and lipid metabolism and the important role of lipid metabolism in trophoblast cells, in this study, we characterized the mechanism by which autophagy induced by the mTOR pathway is involved in PPAR*γ*-mediated regulation of trophoblastic lipid metabolism through exogenous activation and inhibition of PPAR*γ* and autophagy in ovine trophoblastic cells. The work provides valuable insights into lipid metabolism in trophoblasts during pregnancy.

## 2. Material and Methods

### 2.1. Cell Culture and Treatment

All procedures were approved by the Institutional Animal Care and Use Committee (IACUC) of the Shihezi University (A2020-149-01). Sheep trophoblast cells (STCs) were isolated, identified, and preserved in our laboratory [27]. Frozen trophoblast cells were thawed quickly and resuscitated in a 37°C water bath. Cells were cultured with DMEM complete medium (Gibco, Grand Island, NY, USA) containing 10% fetal bovine serum (FBS) (Biological Industries, ISR), 100 U/mL penicillin, and 100 ng/mL streptomycin (Hyclone, South Logan, UT, USA). The resuscitated cells were inoculated into 12-well plates, and culture was continued until cell confluence reached 80% for further studies.

Exogenous drug treatment of STCs involved groups treated with rosiglitazone (RSG) (5 *μ*M), GW9662 (GW) (20 *μ*M) [28], rapamycin (RAPA) (100 nM), 3-methyladenine (3-MA) (5 mM) [29], GW9662 and rapamycin, and 3-MA and rosiglitazone (all reagents from MCE, Monmouth Junction, NJ, USA). For groups treated with pairs of drugs, cells were pretreated with GW9662 or 3-MA for 2 h followed by addition of rapamycin or rosiglitazone, respectively. Cells were collected 12 h, 24 h, and 48 h after drug treatment for follow-up index determination. The final concentration of dimethyl sulfoxide (DMSO) in each group was 0.1%.

### 2.2. Western Blot

The culture medium was decanted after drug treatment, and cells were washed three times with PBS. Total protein was extracted from cells using radioimmunoprecipitation assay buffer (Beyotime, Shanghai, CN) containing 1% PMSF (Boster, Wuhan, CN). The protein concentration in each sample was determined using the BCA Protein Concentration Assay Kit (Beyotime, Shanghai, CN). Samples were adjusted to the same concentration with 1× protein loading buffer. Proteins were separated by sodium dodecyl sulfate-polyacrylamide gel electrophoresis (SDS-PAGE) (Boster, Wuhan, CN) and were then transferred to a 0.22 *μ*m PVDF membrane (Biosharp, Guangzhou, CN) by a semidry transfer method. Membranes were blocked in Tris-buffered saline containing 0.5% Tween-100 with 10% nonfat milk for 2 h and then were incubated with the following antibodies as appropriate overnight at 4°C: anti-p70S6K (1 : 1000, Cell Signaling Technology, MA, USA), anti-phospho-p70S6K (Thr389) (1 : 1000, Cell Signaling Technology), anti-LC3B (1 : 1000, Sigma, St. Louis, MO, USA), anti-Beclin1 (1 : 500, Sigma, St. Louis, MO, USA), or anti-*β*-actin (1 : 1000, Beyotime, Shanghai, CN). Membranes were washed three times with Tris-buffered saline containing 0.5% Tween-100 for 5 min and then incubated with anti-mouse DyLight™ 680 (1 : 15000, Abcam, San Francisco, USA) and anti-rabbit DyLight™ 800 (1 : 30000, Abcam, San Francisco, USA) secondary antibodies at room temperature for 1 h. The membrane was washed, and ImageJ software (National Institutes of Health, Bethesda, MD, USA) was used to analyze the gray values of protein bands and calculate relative protein expression. The *β*-actin protein was used as the internal reference.

### 2.3. Triglyceride Content Test

Treated cells were harvested with a cell scraper and were resuspended in PBS. The supernatant was collected by centrifugation at 1000 g for 10 min and discarded, and the cell precipitate was collected. A mixture of n-heptane and isopropanol (1 : 1; 1 mL) was added followed by ultrasonic crushing for 1 min (intensity 20%, ultrasonic 2 s, and stop 1 s). The lysate was centrifuged at 8000 g at 4°C for 10 min, and the supernatant was retained. Triglyceride (TG) content detection in the supernatant was performed with a triglyceride content detection kit (Sangon Biotech, Shanghai, CN) [30].

### 2.4. Lipid Oxidation Assessed by Malondialdehyde Formation

Cells were collected from 6-well plates and were added to radioimmunoprecipitation assay buffer (100 *μ*L) on ice for 15 min. The supernatant was obtained by centrifugation at 12000 g for 10 min, and 100 *μ*L volumes were used for assessing malondialdehyde (MDA) formation. Standards (100 *μ*L) of different concentrations were used for standard curve preparation, and PBS (100 *μ*L) was used as a control. Two hundred micromoles of MDA test solution was added, and samples were mixed by shaking, followed by heating at 100°C for 15 min. Samples were cooled to room temperature and centrifuged at 1000 g for 10 min. The supernatants (200 *μ*L) were added to a 96-well plate, and absorbance was measured at 532 nm using a microplate reader.

### 2.5. Lipid Droplet and Lysosome Colocalization Fluorescence Staining

STCs were inoculated into 24-well plates, were cultured to 80% confluency, and were then treated with the compounds described above. The medium was discarded, cells were washed three times with PBS, and BODIPY 493/503 (1 : 200 dilution in PBS) lipid droplet (LD) staining solution (Glpbio, Montclair, CA, USA) was added, followed by incubation at 37°C for 20 min in the dark. The BODIPY 493/503 solution was removed, and the cells were washed with PBS three times. Lyso-Tracker Red (Beyotime, Shanghai, CN) staining solution prewarmed to 37°C was added and incubated with the treated cells at 37°C for 10 min. Lyso-Tracker Red was removed and fresh cell culture medium was added. LDs (green) and lysosomes (red) were observed under an inverted fluorescence microscope and photographed. The results were analyzed using ImageJ software.

### 2.6. Statistical Analysis

All data were analyzed statistically using GraphPad Prism V. 8.0 (GraphPad Software, Inc., San Diego, CA, USA). The data were tested for normality and also for homogeneity of variances. One-way ANOVA followed by Tukey's post hoc test and Fisher's LSD was used for multiple comparisons. Data were expressed as mean ± standard deviation. Data for the study came from three separate experiments. Differences were considered statistically significant at *p* < 0.05.

## 3. Results

### 3.1. Induction and Inhibition of PPAR*γ* Impacts Expression of Autophagy-Related Proteins and Modulates Lipid Metabolism in STCs

**Figure 1:**
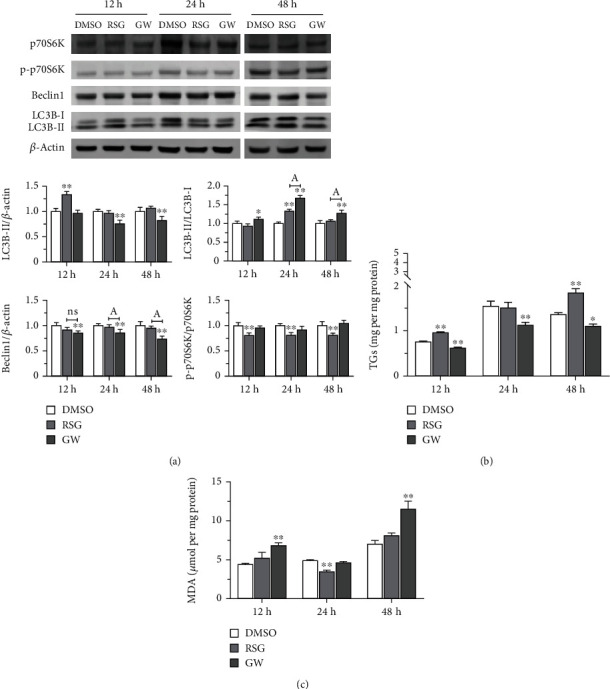
Effects of activation and inhibition of PPAR*γ* activity on autophagy-related proteins and lipid metabolism in trophoblast cells. (a) After activating and inhibiting PPAR*γ* activity by rosiglitazone (RSG) and GW9662 (GW), respectively, Western blot was used to detect the levels of p-p70S6K, p70S6K, Beclin1, LC3B-II, and LC3B-I proteins at 12 h, 24 h, and 48 h. (b) TG content at different timepoints. (c) MDA content as an indicator of lipid oxidation at different timepoints. Data are shown as mean ± standard deviation of three independent trials. ^∗^*p* < 0.05 and ^∗∗^*p* < 0.01.

The roles of PPAR*γ* and the mTOR signaling pathway in lipid metabolism in STCs were examined by exogenous treatment with the PPAR*γ* activator rosiglitazone (RSG) and inhibitor GW9662 (GW) [28]. Phosphorylation of the p70S6K protein is a hallmark of activation by mTOR [31]. The expression of p-p70S6K in STCs was inhibited by RSG treatment at 12 h, 24 h, and 48 h compared with the DMSO group (Figure 1(a)). Beclin1 is an autophagy initiation factor that is involved in the production of lipids that are essential for both autophagy and other membrane trafficking events [32]. Beclin1 protein levels were not significantly different in STCs that were treated with RSG. LC3 is a robust marker of autophagosome formation [33]. The expression level of the LC3B-II protein decreased 12 h after treatment with RSG whereas the ratio of LC3B-II to LC3B-I increased at 24 h (Figure 1(a)). At the 12 h, 24 h, and 48 h timepoints, p-p70S6K protein expression in the GW group did not differ substantially from that of the DMSO group, although Beclin1 expression dropped at all three intervals. The production of the LC3B-II protein decreased 24 h and 48 h after GW exposure, and the LC3B-II : LC3B-I ratio increased at all timepoints (Figure 1(a)). Thus, activation of PPAR*γ* with RSG or inhibition with GW exerts effects both on the mTOR pathway, revealed by alterations in expression of p-p70S6K, and on autophagy, revealed by perturbations in expression of the Beclin1 and LC3 marker proteins in STCs.

Examination of TG content in STCs treated with RSG or GW showed that exposure to the activator significantly increased TG levels after 12 h, whereas TG content decreased significantly after treatment with the inhibitor (Figure 1(b)). After 24 h of drug treatment, TG content in the RSG treatment group was not significantly different to the DMSO group. However, TG levels in cells treated with GW continued to decrease after 24 h. Differences in TG content after RSG or GW treatment were similar at 12 h and 48 h (Figure 1(b)). Thus, activation or inhibition of PPAR*γ* alters TG production in STCs.

The effects of the PPAR*γ* activator RSG and inhibitor GW on cellular lipid oxidation were assessed by testing the formation of MDA. MDA production after RSG exposure decreased significantly after 24 h, but no significant alterations were observed at the 12 h and 48 h timepoints. In contrast, MDA formation increased significantly 12 h and 48 h after treatment with GW, but no significant change was evident at 24 h (Figure 1(c)). These results indicate that stimulating or dampening PPAR*γ* expression impacts lipid oxidation in STCs.

### 3.2. Inhibition of the mTOR Pathway or Autophagy Perturbs Lipid Metabolism in Trophoblast Cells

**Figure 2:**
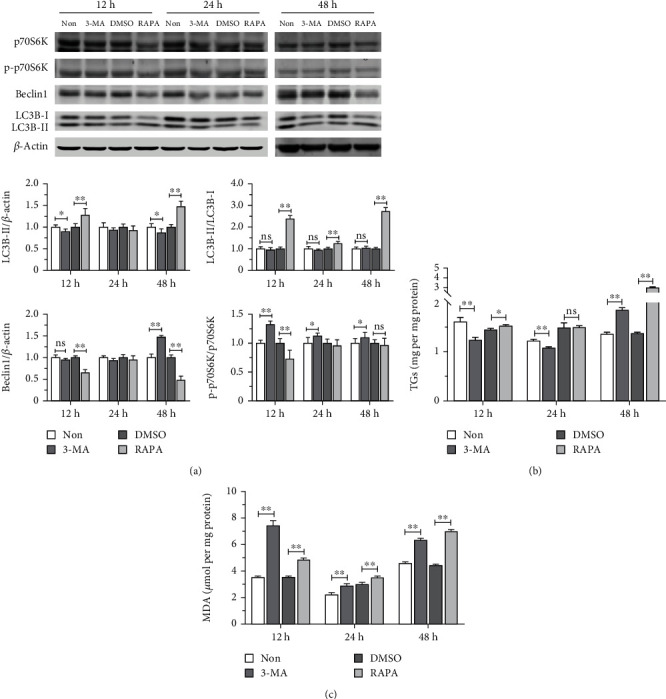
Effects of autophagy activation and inhibition on autophagy-related proteins and lipid metabolism in trophoblast cells. (a) After activating and inhibiting autophagy by rapamycin (RAPA) and 3-MA, respectively, Western blot was used to detect the levels of p-p70S6K, p70S6K, Beclin1, LC3B-II, and LC3B-I proteins at 12 h, 24 h, and 48 h. (b) TG content at different timepoints. (c) MDA content as an indicator of lipid oxidation at different timepoints. Since 3-MA is water-soluble, the control group was also labeled as the nontreatment group (Non) without DMSO. Data are shown as mean ± standard deviation of three independent trials. ^∗^*p* < 0.05, ^∗∗^*p* < 0.01, and ^ns^*p* > 0.05.

In order to explore further the role of autophagy in lipid metabolism in trophoblast cells, the mTOR inhibitor rapamycin and autophagy inhibitor 3-MA were added exogenically to STCs. 3-MA is an inhibitor of PI3K, which is widely used as an inhibitor of autophagy by inhibiting class III PI3K [34]. Western blot results showed that the p-p70S6K : p70S6K ratio increased 12 h, 24 h, and 48 h after 3-MA treatment compared with the negative control group (Figure 2(a)). In contrast, Beclin1 protein expression increased only at the 48 h timepoint. Moreover, expression of the LC3B-II protein decreased 12 h and 48 h after 3-MA treatment. The LC3B-II : LC3B-I ratio did not change significantly throughout the experiment. Rapamycin (RAPA) inhibited the production of p-p70S6K protein at 12 h, decreased Beclin1 expression at both 24 h and 48 h, and increased LC3B-II protein levels at 12 h and 48 h. In addition, the LC3B-II : LC3B-I ratio increased at all three timepoints (Figure 2(a)). These data indicate that inhibitors of the mTOR pathway or autophagy perturb expression of marker proteins for these processes in STCs.

The TG content in STCs exposed to mTOR or autophagy inhibitors was examined. Treatment with 3-MA significantly decreased TG concentrations at the 12 h and 24 h timepoints, but the levels increased after 48 h. In contrast, RAPA treatment increased TG levels significantly at 12 h and 48 h, but no appreciable difference was evident at the 24 h timepoint (Figure 2(b)).

The effects of the mTOR inhibitor rapamycin and autophagy inhibitor 3-MA on cellular lipid oxidation in STCs were assessed by examining the production of MDA. MDA formation increased significantly after 12 h, 24 h, and 48 h of treatment with either RAPA or 3-MA (Figure 2(c)). Thus, lipid oxidation in STCs is perturbed by inhibition of the mTOR pathway or by interfering with autophagy.

### 3.3. Autophagy Induced by mTOR Perturbs PPAR*γ* Regulation of Lipid Metabolism in STCs

**Figure 3:**
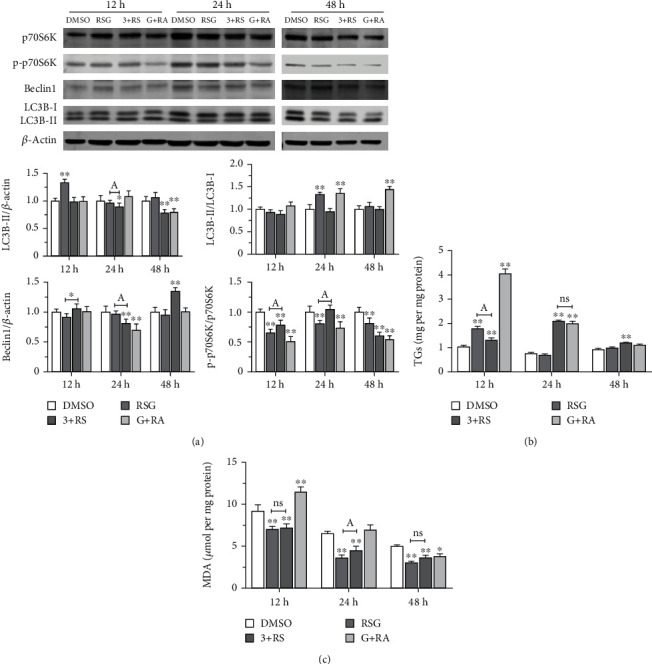
Effect of autophagy induced by mTOR on PPAR*γ* regulation of lipid metabolism in STCs. (a) The levels of p-p70S6K, p70S6K, Beclin1, LC3B-II, and LC3B-I proteins at 12 h, 24 h, and 48 h were detected by Western blot after inhibiting PPAR*γ* activity before blocking mTOR pathway and after inhibiting autophagy before activating PPAR*γ* activity. (b) TG content at different timepoints. (c) MDA content as an indicator of lipid oxidation at different timepoints. Data are shown as mean ± standard deviation of three independent trials. ^∗^*p* < 0.05, ^∗∗^*p* < 0.01, ^A^*p* < 0.05, and ^ns^*p* > 0.05.

To investigate the role of the mTOR pathway in PPAR*γ*-mediated regulation of lipid metabolism in trophoblast cells, the pathway was blocked by RAPA after exogenous inhibition of PPAR*γ* activity with GW9662 (GW+RAPA). In addition, STCs were treated first with 3-MA and subsequently with RSG to activate PPAR*γ* (3-MA+RSG). In comparison to cells treated just with RSG, Western blot results demonstrated that cells treated with 3-MA+RSG significantly raised the ratio of p-p70S6K : p70S6K at 12 h and 24 h. Moreover, Beclin1 protein expression level increased at 12 h and 48 h but decreased at 24 h. The expression level of LC3B-II protein decreased at all three time intervals. The LC3B-II : LC3B-I ratio decreased only at 24 h. Compared with cells treated with DMSO, the p-p70S6K : p70S6K ratio decreased at the 12 h, 24 h, and 48 h timepoints in cells treated with GW+RAPA. Moreover, Beclin1 expression was inhibited at 24 h, and expression of LC3B-II was reduced at 48 h. In addition, the LC3B-II : LC3B-I ratio increased significantly at 24 h and 48 h under these conditions whereas this ratio decreased only at 24 h (Figure 3(a)). The results reveal the interplay between autophagy, the mTOR pathway, and PPAR*γ* expression in STCs.

TG content in STCs treated with RSG, 3-MA+RSG, or GW+RAPA increased significantly at 12 h after treatment. However, TG levels in cells treated with both 3-MA and RSG were reduced compared with cells treated only with RSG (Figure 3(b)). Moreover, TG content in STCs treated either with 3-MA+RSG or with GW+RAPA increased significantly, and there was no difference between the RSG and DMSO groups after 24 h. After 48 h of drug treatment, TG levels in cells exposed to 3-MA and RSG increased significantly, whereas no changes were apparent in other treatment groups (Figure 3(b)). Thus, TG levels in STCs are perturbed either when the mTOR pathway is blocked by RAPA after exogenous inhibition of PPAR*γ* activity with GW or when autophagy is inhibited with 3-MA and PPAR*γ* subsequently is activated with RSG.

Compared with the DMSO group, MDA levels indicative of lipid oxidation in cells treated with RSG only or with both 3-MA and RSG decreased significantly after 12 h of treatment. Moreover, MDA levels in STCs treated with GW9662 and RAPA increased significantly after 12 h. Lipid oxidation in RSG-treated STCs and in cells exposed to both 3-MA and RSG decreased further compared to the DMSO group after 24 h (Figure 3(c)). However, MDA levels in cells treated with both 3-MA and RSG were elevated significantly compared to cells treated only with RSG. After 48 h of drug administration, lipid oxidation decreased significantly in cells treated with drugs, and there was no significant difference between the RSG group and 3-MA+RSG group (Figure 3(c)). These results indicate that lipid oxidation in STCs is perturbed both when the mTOR pathway is blocked after inhibition of PPAR*γ* activity and when autophagy is inhibited and PPAR*γ* subsequently is activated.

### 3.4. Fluorescence Staining Reveals That Interference with the mTOR Pathway Modulates Lipid Droplet and Lysosome Concentrations in STCs

**Figure 4:**
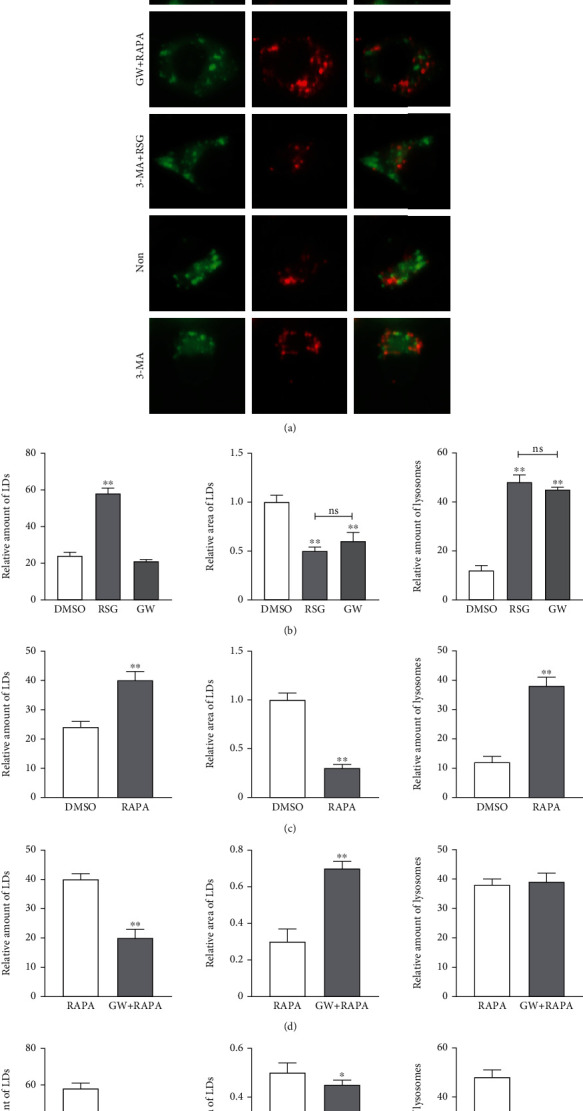
Colocalization of lipid droplets and lysosomes. (a) The lipid droplets and lysosomes in each group were colocated by fluorescence staining with BODIPY 493/503 and Lyso-Tracker after drug treatment for 12 h. Scale bars, 20 *μ*m. (b–f) The amount and area of LDs and lysosomes were quantified using ImageJ. Data are shown as mean ± standard deviation of three independent trials. ^∗^*p* < 0.05, ^∗∗^*p* < 0.01, and ^ns^*p* > 0.05.

In view of the diverse temporal effects of interference with autophagy and lipid metabolism that were described in the preceding sections, the colocalization of LDs and lysosomes was examined in STCs only 12 h after treatment with drugs. The amount of lysosomes and LDs increased significantly, lysosomes and LDs were colocalized, and the average LD area decreased significantly after RSG treatment (Figure 4(a)). In contrast, the amount of LDs did not change significantly, but the area decreased after GW treatment (Figure 4(b)). Exogenous addition of RAPA inhibits mTOR activity to promote autophagy, or 3-MA directly blocks autophagy as outlined above. LD levels increased, the LD area decreased, and the number of lysosomes increased after promoting autophagy (Figure 4(c)). Compared with the negative control group, the area and amount of LDs were significantly reduced, and the amount of lysosomes was increased after inhibition of autophagy (Figure 4(f)). Furthermore, the amount of LDs in the GW+RAPA group decreased, but the area increased, while the number of lysosomes did not change, compared with the RAPA group (Figure 4(d)). Finally, the amount and area of LDs and lysosomes in cells treated with both 3-MA and RSG showed a downward trend compared with the RSG group (Figure 4(e)). Overall, these results indicate that the amount of LDs and lysosomes in STCs is impacted by perturbation of the mTOR pathway, autophagy, and/or PPAR*γ* expression levels.

## 4. Discussion

Strict regulation of lipid homeostasis depends on the balance between lipid intake and lipid degradation which is determined by multiple factors and mechanisms. The PPAR*γ* signaling pathway is a major signaling process that regulates fat metabolism and is at the hub of the signaling pathway regulation network that is involved in the regulation of adipocyte differentiation and fat deposition [35]. Increased PPAR*γ* expression promotes the enhanced expression of downstream target genes, including the genes for fatty acid binding protein 4 (FABP4) and apolipoprotein C-1 (APOC1) which is consistent with the induction of intracellular LD formation [36]. *Mycobacterium bovis* BCG infection induces the expression of PPAR*γ* and its downstream target genes in macrophages, and the PPAR*γ* antagonist GW9662 inhibits BCG-induced lipid accumulation [37]. Moreover, stimulation of PPAR*γ* with the ligand pioglitazone in human placental trophoblast cells and goat mammary glands upregulated the transcription levels of fat metabolism-related genes FABP4 and the perilipin-2 (PLIN2) protein which promotes fat deposition [38]. The results presented in the current study also indicate that exogenous addition of rosiglitazone promotes lipid deposition in STCs, while the addition of GW9662 exerts the opposite effect. However, this pattern varied with time of exposure to the ligands. Meanwhile, the MDA levels in STCs changed from low to high or vice versa. We speculate that a steady-state regulator is degraded when lipid concentrations accumulate to a threshold level and that stored lipids are then utilized to maintain lipid homeostasis.

Autophagy involves protective self-digestion of intracellular organelles in response to stress and is necessary to maintain cellular homeostasis. Autophagy plays an important role in improving lipid metabolism [39]. The activation of PPAR*γ* and the initiation of autophagy seem to be interrelated in diverse cell types, and PPAR*γ* and autophagy synergistically regulate lipid metabolism. Activation of PPAR*γ* induces autophagy whereas its inhibition reduces autophagy [40]. Upregulation of PPAR*γ* activates AMPK/mTOR-dependent autophagy [41]. AMPK is an important regulator of autophagy and is essential for phagosome-lysosome fusion [42]. PPAR*γ* inhibitor GW9662 activates the AMPK pathway in mouse macrophages, thereby regulating autophagy [43]. In addition, deletion of the gene for PPAR*γ* in bone marrow mesenchymal stem cells significantly increased phosphorylation of p70S6K (p-p70S6K) which is a major downstream effector of the mTOR signaling pathway [44]. In this study, it was also found that activation of PPAR*γ* inhibited mTOR activity and induced autophagy in STCs. In order to explore further the role of autophagy in lipid metabolism of trophoblastic cells, we modulated autophagy by adding rapamycin and 3-MA exogenically. Lipid accumulation was observed after autophagy was promoted within a certain time period, whereas lipid accumulation decreased after autophagy was inhibited. Similarly, increased MDA levels were observed after inhibition of autophagy which suggests that cells may hydrolyze lipids through nonautophagy pathways to maintain homeostasis after autophagy inhibition.

Rapamycin inhibits the proliferation and differentiation of 3T3-L1 cell lines and human precursor adipocytes by blocking PPAR*γ* activity [45]. The mTOR protein positively regulates the activity of PPAR*γ* to promote lipid synthesis and deposition [46]. Therefore, the epistatic relationship between PPAR*γ* and mTOR may be environment-dependent, or PPAR*γ* may be activated by mTOR and operate a negative feedback loop to limit mTOR activation [47]. We found here that inhibition of PPAR*γ* activity before blocking the mTOR pathway induced autophagy and promoted lipid accumulation by STCs within certain time periods. Compared with activating PPAR*γ*, inhibition of autophagy may reduce the lipid accumulation ability of STCs which suggests that PPAR*γ* may affect lipid accumulation through mTOR-mediated autophagy.

Autophagy regulates lipid metabolism either by inducing lysosomal lipolysis of LDs (liphagy) or by participating in the formation of LDs [48]. In addition to its role in LD catabolism, several recent studies have shown that autophagy is involved in the formation of LDs by providing fatty acids for the synthesis of triacylglycerol. Triacylglycerol is a key source of calories for energy homeostasis. Deactivation of the autophagy pathway resulted in a significant decrease in triacylglycerol levels in mature seeds of Arabidopsis [49]. The results of this study indicate that autophagy is more likely to participate in the formation of LDs in trophoblastic cells.

Autophagy-related genes (ATGs) are implicated in the formation of LDs [50]. For example, the MAP1-LC3 coupling system that plays a central role in autophagy is essential for LD formation in hepatocytes [51]. The LC3 protein is expressed in most cell types, and, as LC3-II is a structural component of mature autophagy, the protein is often used as a specific marker for autophagy. ATGs are involved in the formation of LDs both in *Caenorhabditis elegans* and in cultured mammalian cells [52]. The ATG5 protein exerts a key role in lipid metabolism by regulating LD formation in the kidney [53]. These findings suggest that a division of labor occurs between ATGs that dictates which autophagy pathway is redirected to address the effects of lipid degradation or accumulation. The Beclin1 (ATG6) protein differs from other ATGs due to its nonautophagy specificity. This protein forms part of the lipid kinase complex that is involved in cytoplasmic lipid metabolism [54]. Previous studies have shown that Beclin1 has the potential to promote lipid storage in cells [32, 55]. We observed here that LC3B-II (ATG8) protein levels often increased and decreased in the cells with increased and decreased TG content, respectively. In parallel, the lipid content and Beclin1 protein levels decreased in a time-dependent manner after inhibition of PPAR*γ* activity.

LDs are transported to lysosomes through autophagy which suggested the existence of a lysosomal acid lipase that hydrolyzed lipid droplets [56]. Antagonists of PPAR*γ* promote autophagy of microglial cells, thereby enhancing the formation of microglial autophagosomes and the degradation of lysosomes [57]. Moreover, inhibition of PPAR*γ* reduced hepatic steatosis in mice fed with a high-fat diet [58]. *In vivo* injection of the autophagy inhibitor chloroquine in mice blocked high-fat diet-induced obesity and inhibited autophagy levels of 3T3-L1 preadipocytes *in vitro* which promoted proteasome-dependent degradation of PPAR*γ* and weakened lipid differentiation. Therefore, activated autophages increase the stability of PPAR*γ* and promote lipid differentiation [59]. In order to clarify the links between the expression of PPAR*γ*, autophagy, lysosome, and LDs in STCs, the colocalization of LDs and lysosome in the treatment group was detected here in STCs 12 h after drug administration. The numbers of lysosomes and LDs increased after the stimulation of PPAR*γ* activity, but the area of LDs decreased. Combined with the colocalization of LDs and lysosomes, we speculate that LDs are decomposed into smaller packages. This process is also facilitated by the induction of autophagy with rapamycin. Further exploration revealed that, compared to cells activated for PPAR*γ*, cells in which PPAR*γ* was activated after inhibition of autophagy contained decreased numbers of lysosomes due to inhibition of autophagy. The amount and area of LDs also showed downward trends. Conversely, compared with autophagy induction only, PPAR*γ* activity was inhibited before autophagy was promoted by rapamycin, and the concentration of LDs in cells decreased although the LD area increased.

## 5. Conclusions

**Figure 5:**
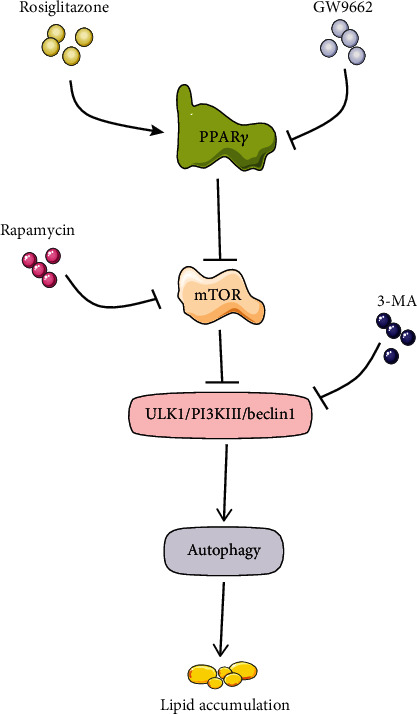
PPAR*γ* is involved in the regulation of lipid accumulation in STCs through mTOR pathway-mediated autophagy. PPAR*γ* may inhibit the activity of mTOR in a time-dependent manner, thereby inducing autophagy and promoting lipid accumulation which provides the energy basis for rapid proliferation of trophoblast cells during conceptus prolongation.

In summary, our results suggest that PPAR*γ* promotes autophagy by inhibiting the activity of the mTOR pathway, thereby promoting an increase in lysosome numbers and LD accumulation (Figure 5). We put forward a new idea that the effect of PPAR*γ* on the lipid metabolism of trophoblast cells is in a dynamic process; that is, PPAR*γ* promotes autophagy while promoting lipid accumulation, so that the cells are in a dynamic process of lipid metabolism, which may provide lipid source and energy basis for the rapid proliferation of trophoblast cells during embryo implantation.

## Data Availability

The datasets used during the current study can be obtained from the corresponding authors upon request.
